# Validation and calibration of the Food Consumption Frequency Questionnaire for pregnant women

**DOI:** 10.1590/1516-3180.2023.0059.R2.190523

**Published:** 2023-10-09

**Authors:** Sheila Monteiro Brito, Jerusa da Mota Santana, Marcos Pereira, Djanilson Barbosa Santos, Ana Marlucia Oliveira

**Affiliations:** 1 MSc, PhD. Adjunct Professor, Health Care Practices: Nutrition, Health Sciences Center, Universidade Federal do Recôncavo da Bahia (UFRB), Santo Antônio de Jesus (BA), Brazil.; 2 MSc, PhD. Adjunct Professor, Health Care Practices: Nutrition, Health Sciences Center, Universidade Federal do Recôncavo da Bahia (UFRB), Santo Antônio de Jesus (BA), Brazil.; 3 MSc, PhD. Adjunct Professor, Department of Public Health, Institute of Collective Health, Universidade Federal da Bahia (UFBA), Salvador (BA), Brazil.; 4 MSc, PhD. Adjunct Professor, Collective Health, Health Sciences Center, Universidade Federal do Recôncavo da Bahia (UFRB), Santo Antônio de Jesus (BA), Brazil.; 5 MSc, PhD. Full Professor, School of Nutrition, Universidade Federal da Bahia (UFBA), Salvador (BA), Brazil.

**Keywords:** Eating, Validation studies [publication type], Pregnancy, Prenatal care, Dietary patterns, Food consumption frequency questionnaire, Calibration

## Abstract

**BACKGROUND:**

Few food frequency questionnaires (FFQ) have been validated for pregnant women, particularly those in small- and medium-sized cities in different regions of Brazil.

**OBJECTIVES:**

To validate and calibrate a semiquantitative FFQ for pregnant women.

**DESIGN AND SETTING:**

The study was validated with a sample of 50 pregnant women (≥ 18 years) enrolled in Brazilian prenatal services.

**METHODS:**

An FFQ and a 24-hour recall were used to evaluate dietary intake. Dietary variables were tested for normality and log-converted when asymmetrical. Pearson's Correlation Coefficient was used to validate the questionnaire. Linear regression was applied to extract calibration factors. All variables underlying the consumption analysis were adjusted for energy.

**RESULTS:**

The mean age of the pregnant women was 26 years ± 6.2 years; 58% were in their first trimester, and 30% were identified as overweight/obese. The Pearson correlation analysis results indicated that the FFQ overestimated energy and nutrient intake, whose coefficients ranged from −0.15 (monounsaturated fat) to 0.50 (carbohydrate). Adjusting for energy reduced the mean values of intake coefficients, which now ranged from −0.33 (sodium) to 0.96 (folate). The calibration analysis results indicated variation in the coefficients from −0.23 (sodium) to 1.00 (folate). Calibration produced satisfactory coefficients for the FFQ compared with the reference standard for energy, macronutrients, monounsaturated fat, cholesterol, vitamins B12/C, folate, sodium, iron, and calcium.

**CONCLUSIONS:**

After validating and calibrating tests, we observed that the FFQ was adequately accurate for assessing the food consumption of the pregnant women in this study.

## INTRODUCTION

Currently, researchers in nutritional epidemiology have made efforts to identify evaluation methods and analysis techniques to obtain precise and accurate food consumption data during different life stages, considering cultural conditions, the complexity of factors associated with human food, and peculiarities of regional and local contexts. This task requires the development of methodological instruments for qualitative-quantitative assessments to understand the role of food and nutrients in the occurrence of health- and disease-related events.^1^

Instruments available to assess food consumption are likely to have measurement errors, producing biased dietary intake estimates.^2–4^ Among these instruments, the 24-hour Recall Questionnaire (24hR) and the Food Frequency Questionnaire (FFQ) are widely employed in population studies in their quantitative or semi-quantitative versions. While the 24hR characterizes the food and beverage consumption in the 24 hours before the interview,^2–4^ the FFQ assesses an individual's customary diet in a specific period. One advantage of these methods is their low cost, which allows the assessment of a more significant number of individuals, thereby enabling effective association of dietary patterns with outcomes of interest. Thus, these instruments have been used to estimate risk trends for the consumption of nutrients per degree of exposure to different intake levels.^1,5–7^

The FFQ, specifically, is widely used to evaluate the dietary habits and consumption patterns of people from different sociocultural and economic contexts.^8,9^ In the absence of a gold standard method to achieve these goals, existing methods should be adapted and validated for specific populations to understand food consumption patterns and reliably minimize associated errors. This validation involves comparing the nutrient intake estimates obtained by the test method with those of a standard, using different statistical analyses.^2,10^ Furthermore, calibrating the instrument is essential to reduce or eliminate bias in the underestimation or overestimation of nutrient intake estimates and obtain new intake parameters closer to the benchmark.^2^

However, the validated FFQs available for specific Brazilian population groups are mainly aimed at adults living in large urban centers.^3,10,11^ Few instruments have been validated for pregnant women, particularly those in small- and medium-sized cities in different regions of the country.^12^

A precise and accurate assessment of food consumption is relevant, specifically during pregnancy, because inadequate nutrient intake during pregnancy is a risk factor in the development of morbimortality and occurrence of chronic diseases in mothers and children in the long term.^13^

## OBJECTIVE

This study aimed to validate and calibrate a semi-quantitative FFQ, for pregnant women receiving primary care in a municipality in Brazil's Northeast region.

## METHODS

### Study design and sample

This validation and calibration study of a food frequency method is nested in the research project “*Pregestational and gestational risk factors for postpartum maternal weight retention in a municipality in the Recôncavo Baiano*” undertaken by researchers from the Federal University of Recôncavo da Bahia.

This study adopted 24hR as the reference standard,^3–5^ and the FFQ method was validated. A convenience sample of 53 pregnant women enrolled in prenatal care in 2012 in a Northeast municipality was selected. This sample size complies with the recommendation of 50–100 participants.^6,14,15^ Three pregnant women were excluded because they had outlier values for total energy (above 6,000 kcal) in the 24hR, which could increase the possibility of a biased interpretation of other nutrients’ intake values.^16^

Data were collected between February and December 2012 by researchers adequately trained in nutrition in the municipal health units during the first prenatal care visit. We gathered information on demographics (maternal age), socioeconomic status (schooling, income, marital status, and employment status), health (pathological history and clinical complications), reproductive history (gestational age, parity, and interpartum interval), and anthropometric characteristics, including lifestyle habits (alcohol consumption, smoking habits, and physical activity).

### Food-frequency questionnaire development and analysis

The customary food consumption pattern of these pregnant women was captured using the FFQ, including information on the time and place of meals, type of preparation, and amount of food consumed. This instrument comprised a list of seventy-three foods, selected based on information from a pilot study's 24hR. Evidence indicates that the inclusion of 60–130 food items in an FFQ is sufficient to characterize an individual's usual diet.^17^

A minimum consumption frequency of 15% for each food item identified in the pilot study was adopted as an inclusion criterion for creating the FFQ.^18^ Thus, 19 items were excluded from the frequency list: whole-grain rice, pasta, rye bread, polenta, chicory, zucchini, green beans, hamburgers, shrimp, pizza, mayonnaise, ice cream, chocolate bars, French fries, pears, grapes, canned fish, pudding, and wine. The following were included in the list: cassava, eggplant, oats, couscous, plantain, tangerine, guava, ready-made sauce, concentrated broth, ready-to-eat soup, jerked beef, sun-dried meat, and bologna.

Regardless of the criterion previously established, some regional foods representative of the culture and eating habits, whose consumption is related to seasonal variation, were also included in the list: *beiju* (tapioca pancake), *andu* (type of bean), and breadfruit. Sixteen items were thus included in the final list.

The qualitative-quantitative information on the frequency of food consumption, retrospective to the month before the interview,^19^ was stratified into the following categories: more than three times a day, two to three times a day, once a day, five to six times a week, two to four times a week, once a week, and one to three times a month.

Images of the portions and utensils used were captured in a photographic record album and used to obtain the standard serving size for each food.^20^ This strategy was used to reduce errors in estimating the actual amount of food consumed by the respondent.

All reported frequencies were converted into daily frequencies to analyze the consumption data. For this conversion, we considered the number of times the food was consumed per day and multiplied by the value “1” whenever the food was consumed daily. The mean reported interval was estimated and then divided by seven (weekly consumption) and 30 (monthly consumption) to calculate the daily frequency from weekly or monthly consumption. Thus, all consumption was expressed as mean daily consumption.

The food consumption measurement unit in grams per day was standardized based on the food composition table^21^ and the list of replacement food groups in the food pyramid for the Brazilian population.^7^ Excel 2010 (Microsoft, Washington, United States) and the Brazilian Food Composition Table^21^ were used to estimate the daily values of energy and nutrients in the diet according to the FFQ record. We used Virtual Nutri Plus software (University of São Paulo-USP, São Paulo, Brazil) to evaluate the data obtained from the 24hR.

### Statistical analysis

Statistical analyses were performed considering the following steps:

- Test of normality of dietary variables: Dietary variables (macronutrients and micronutrients) were tested for normality (Shapiro-Wilk test) to assess compliance with the method's assumptions. To improve their normality, the variables were log-converted when the normality assumption was not met.
- Comparison between the mean differences in caloric and nutrient availability measured by the two instruments (FFQ and 24hR): We employed the paired t-test for these analyses.
- Comparison between the correlation coefficients of the crude values of energy, macronutrients, and micronutrients estimated by the FFQ and 24hR: We used Pearson's correlation coefficient to observe the agreement between the values estimated by these methods.
- Adjustment for energy: The estimated values of the dietary variables were adjusted for energy, to minimize the effect of total caloric intake on the number of nutrients in the diet. For this, we employed residual analysis of linear regression.^1,3^
- Validation analysis: Validation analysis was performed using Pearson's correlation test to compare the correlation coefficients of nutrients, estimated by the FFQ and 24hR and adjusted in the previous analysis stage.
- Calibration analysis: Finally, a calibration analysis was performed to minimize and remove errors in the instrument under test (FFQ) by applying a linear regression technique between the adjusted and validated nutrient values of the FFQ and the adjusted nutrients of the 24hR.
- Comparison between energy and nutrient estimates from the calibrated FFQ and energy-adjusted 24hR estimates, using Pearson's correlation coefficient: This analysis aimed to verify the agreement between the final estimates obtained by the test method and reference methods.

We employed SPSS version 17.0 (Chicago, United States) for statistical analyses. The statistical significance level of P ≤ 0.05 was chosen for the acceptance of the test's significance.

### Ethical approval

This study was approved by the Ethics Committee for Research Involving Human Beings of the Faculdade Adventista da Bahia (No. 4369.0.000.070-10) on September 14, 2010. All study procedures abided by the Code of Ethics of the World Medical Association (Declaration of Helsinki) for experiments involving humans. Informed consent was obtained for experimentation with human participants and their privacy rights were respected.

## RESULTS

### Description of participants

The maternal sociodemographic, obstetric, and anthropometric characteristics are presented in Table 1. Most pregnant women (90%) were aged less than 35 years (mean = 26 years, standard deviation [SD] = 6.2 years). The level of schooling up to high school was 96.0%, and income ≤ 1 MW was reported by 32.0% of households. The Catholic religion was adopted by 50% of pregnant women; marriage or common-law marriage was reported by 82%; self-declared ethnicity/skin color was Black for 48%; smoking was reported by 60%; and alcohol use was reported by 34%.

**Table 1:** Sociodemographic, obstetric, and anthropometric characterization of pregnant women. Santo Antônio Jesus (Bahia), Brazil, 2012

Variables		n (50)	%
**Maternal age**			
	< 35 years	45	90
	≥ 35 years	5	10
**Maternal schooling**			
	< High School	48	96
	≥ High School	2	4
**Income**			
≤ 1 minimum wage		16	32.0
> 1 minimum wage		34	68.0
**Religion**			
	Catholic	26	51
	Protestant	19	38.8
	No religion	5	10.2
**Marital status**			
	Single	9	18
	Married/living with a partner	41	82
**Ethnicity/skin color**			
	White	4	8
	Brown	20	40
	Black	24	48
	Indigenous	2	4
**Tobacco use**			
	Smoker/former smoker	30	60
	Non-smoker	20	40
**Alcohol use**			
	Yes	17	34
	No	33	66
**Gestational trimester**			
	First	29	58
	Second	11	22
	Third	10	20
**Prenatal care visits**			
< 7 visits		46	91.8
≥ 7 visits		4	8.2
**Number of pregnancies**			
	Primiparous	32	64
	Multiparous	18	36
**Gestational complications**			
	Yes	30	60
	No	20	40
**Maternal height**			
	≤ 150 cm	4	8.3
	> 150 cm	46	91.7
**Pregestational anthropometric status**			
	Underweight	10	20
	Eutrophy	25	50
	Overweight	12	24
	Obesity	3	6
**Gestational anthropometric status**			
	Underweight	10	20
	Eutrophy	19	38
	Overweight	13	26
	Obesity	8	16

Approximately 58% of the participants were included in the study in the first gestational trimester, and primiparity was 64%. We found that 91.8% of pregnant women made fewer than seven prenatal care visits, and 60% reported pregnancy complications.

The mean height was 160 cm (SD = 0.9 cm), and a height of > 150 cm was observed in 91.7% of cases. The prevalence of pregestational eutrophy was 55.1% (24.3 kg/m^2^, SD = 14.2 kg/m^2^) and overweight (overweight/obesity) was 40% (27.7 kg/m^2^, SD = 15.3 kg/m^2^). A mean weight gain of 5 kg was recorded (SD = 6.4 kg) during the gestational period, and 30% of the pregnant women were overweight/obese.

The descriptive analysis results indicated that carbohydrates, total fat, saturated fat, polyunsaturated fat, fiber, folate, vitamin B6, vitamin E, potassium, sodium, magnesium, and zinc from the 24hR did not show a normal distribution and were thus log-converted. Regarding the FFQ, most nutrients were log-transformed, except for vitamin D and monounsaturated fat, which showed a normal distribution.

Table 2 presents the mean values of calories and nutrients from the FFQ and 24hR. The mean difference between the 24hR and FFQ values for energy and most nutrients was statistically significant (P ≤ 0.05) (Table 2). Comparing the estimated values for the mean intake of energy and nutrients recorded by the 24hR and FFQ, we found that the FFQ overestimated the values of caloric availability; macronutrients; vitamins C, E, B3, B5, B6, B12, and folate; and minerals phosphorus, potassium, iron, magnesium, zinc, and selenium. The mean values of the consumption of other nutrients estimated using the two methods were similar (P > 0.05).

**Table 2:** Mean, standard deviation, and difference in means of energy and nutrient intake adjusted for energy from the food frequency questionnaire and 24-hour recall. Santo Antônio de Jesus (Bahia), Brazil, 2012

Nutrient	FFQ		24hR		Difference of means	P value
	Mean	SD	Mean	SD		
Carbohydrate (g)	449.96	165.25	320.13	128.19	6.422	0.000**
Protein (g)	99.59	49.75	78.20	41.50	2.506	0.016*
Fat (g)	85.52	48.42	62.38	30.97	3.004	0.004**
Fibers (g)	34.08	16.23	16.31	9.30	7.328	0.000**
Polyunsaturated fat (g)	15.46	9.52	8.20	9.32	3.952	0.000**
Monounsaturated fat (g)	29.84	20.76	10.82	5.41	6.055	0.000**
Saturated fat (g)	27.89	16.30	18.45	9.92	3.628	0.001**
Cholesterol (mg)	360.70	236.84	289.70	595.55	0.799	0.428
Vitamin A (RE)	4017.09	3302.61	3653.33	13514.42	0.242	0.810
Vitamin D (mcg)	3.58	2.37	11.76	35.76	-1.601	0.116
Vitamin C (mg)	529.19	362.68	130.54	126.39	7.831	0.000**
Vitamin B3 (mg)	31.59	15.97	20.94	10.32	4.167	0.000**
Vitamin B1 (mg)	2.88	1.12	3.06	8.42	-0.151	0.880
Folate (mcg)	494.14	226.41	175.13	332.85	5.786	0.000**
Vitamin B2 (mg)	3.20	1.62	4.31	14.15	-0.559	0.579
Pantothenic acid (mg)	8.23	3.54	4.78	10.62	2.331	0.024*
Vitamin B6 (mg)	3.04	1.56	1.57	2.93	3.786	0.000**
Vitamin E (mg)	26.70	16.55	10.11	11.60	6.991	0.000**
Vitamin B12 (mcg)	28.96	31.18	14.59	38.15	2.246	0.029**
Potassium (mg)	5147.88	2100.00	2212.27	1030.63	10.562	0.000**
Sodium (mg)	2454.76	1348.55	2292.52	2499.57	0.373	0.711
Phosphorus (mg)	1414.30	595.70	1119.80	697.92	2.472	0.017**
Calcium (mg)	919.31	445.90	758.90	556.26	1.858	0.069
Iron (mg)	21.68	10.50	15.93	19.01	2.238	0.030*
Magnesium (mg)	389.35	146.95	191.23	82.86	10.013	0.000*
Copper (mg)	2.90	1.68	1.96	5.66	1.229	0.225
Zinc (mg)	12.85	7.70	8.49	7.41	2.950	0.005**
Selenium (mcg)	138.17	70.78	58.16	72.03	5.959	0.000**
Energy (kcal)	2959.41	1098.64	2154.79	840.78	5.286	0.000**

FFQ = food frequency questionnaire; 24hR = 24-hour recall; SD = standard deviation; RE = retinol equivalent.

* Paired *t*-Test: Comparison of means between FFQ and R24h; P < 0.05;

** P < 0.01.

In the validation analysis, we observed that Pearson's correlation coefficients, obtained by comparing the crude values estimated by the FFQ and 24hR methods, ranged from −0.15 (monounsaturated fat) to 0.50 (carbohydrate). Significant correlations were observed for calories (r = 0.41), carbohydrates (r = 0.55), vitamin E (r = 0.33), potassium (r = 0.37), copper (r = 0.29), iron (r = 0.36), and magnesium (r = 0.37) (Table 3).

**Table 3:** Pearson's correlation coefficient for crude energy and nutrients, adjusted for energy and calibrated, estimated using the food frequency questionnaire and 24-hour recall in a population of pregnant women. Santo Antônio de Jesus (Bahia), Brazil, 2012

Nutrient	Crude *r*	Adjusted *r*	Calibrated *r*
Carbohydrate (g)	0.50*	0.23	0.89**
Protein (g)	0.24	0.08	0.53**
Fat (g)	0.15	-0.05	-0.60**
Fibers (g)	0.17	0.02	0.02
Polyunsaturated fat (g)	0.13	0.03	0.03
Monounsaturated fat (g)	-0.15¨	-0.23	- 0.95**
Saturated fat (g)	0.09	-0.04	0.04
Cholesterol (mg)	0.18	0.01	0.81**
Vitamin A (RE)	0.09	0.18	0.17
Vitamin D (mcg)	-0.12¨	0.21	- 0.18
Vitamin C (mg)	0.31	0.75*	0.73**
Vitamin B3 (mg)	0.28*	0.16	0.15
Vitamin B1 (mg)	0.12	-0.04	0.11
Folate (mcg)	0.05	0.96*	0.92**
Vitamin B2 (mg)	0.14	0.06	0.03
Pantothenic acid (mg)	0.22	0.09	0.03
Vitamin B6 (mg)	0.23	0.08	0.08
Vitamin E (mg)	0.25	0.15	0.15
Vitamin B12 (mcg)	0.13	0.22	0.99**
Potassium (mg)	0.32*	0.24	0.23
Sodium (mg)	-0.14	-0.33*	0.31*
Phosphorus (mg)	0.24	0.10	0.06
Calcium (mg)	0.23	-0.05	- 0.71**
Iron (mg)	0.21	0.11	0.75**
Magnesium (mg)	0.31*	0.18	0.17
Copper (mg)	0.31*	0.20	0.16
Zinc (mg)	0.09	-0.03	0.03
Selenium (mcg)	0.23	0.07	0.23
Energy (kcal)	0.39*	0.01	- 0.38**

RE = retinol equivalent.

Nutrients with normal distribution; others were log-transformed;

* P < 0.05;

** P < 0.01.

When adjusting nutrients for energy, correlation values for most nutrients changed, ranging from reductions (-0.33 for sodium) to increases (0.96 for folate). The correlations increased for vitamins D, B3, B12, C, and folate and were significant for the last two. A significant negative correlation was observed with sodium levels after adjusting for energy (Table 3). After calibration, we noted that the values of the nutrient correlation coefficients of the FFQ and the 24hR ranged from −0.95 (monounsaturated fat) to 0.99 (vitamin B12); were positive and significant for carbohydrate, protein, cholesterol, vitamin C, folate, vitamin B12 and iron; and significant, however, negative for total fat, monounsaturated fat, and energy (Table 3).

Table 4 displays the calibration results, regression coefficients, and respective confidence intervals for the dietary variables adjusted for energy. Variation was observed in the values of calibration factors from −0.23 (sodium) to 1.00 (folate). Calibration results for vitamin C, folate, sodium, phosphorus, and selenium were statistically significant. Table 5 presents the mean values of estimated, residual, constant, and adjusted macronutrients and micronutrients.

**Table 4:** Calibration regression coefficients (α and λ) for energy-adjusted dietary variables, estimated using the food frequency questionnaire and 24-hour recall in a population of pregnant women. Santo Antônio de Jesus (Bahia), Brazil, 2012

Nutrients	α	95% CI		λ	95% CI	
		Minimum	Maximum		Minimum	Maximum
Carbohydrate (g)	2.25	1.96	2.54	0.09	-0.02	0.20
Protein (g)	1.76	1.48	2.03	0.05	-0.09	0.19
Fat (g)	1.79	1.52	2.07	-0.02	-0.17	0.12
Fibers (g)	1.12	0.70	1.54	0.02	-0.26	0.30
Polyunsaturated fat (g)	0.66	0.19	1.13	0.04	-0.37	0.45
Monounsaturated fat (g)	12.51	9.98	15.04	-0.06	-0.12	0.01
Saturated fat (g)	1.24	0.87	1.62	-0.04	-0.31	0.23
Cholesterol (mg)	1.89	0.96	2.82	0.13	-0.24	0.51
Vitamin A (RE)	2.41	0.77	4.06	0.12	-0.36	0.59
Vitamin D (mcg)	3.61	2.81	4.41	0.05	-0.02	0.11
Vitamin C (mg)	1.73	1.64	1.83	0.01	0.01	0.01
Vitamin B3 (mg)	1.40	1.28	1.52	0.01	0.01	0.01
Vitamin B1 (mg)	0.44	0.39	0.49	0.01	-0.01	0.01
Folate (mcg)	0.00	-0.18	0.17	1.00	0.91	1.09
Vitamin B2 (mg)	0.45	0.39	0.52	0.01	0.01	0.01
Pantothenic acid (mg)	0.87	0.82	0.93	0.01	-0.01	0.01
Vitamin B6 (mg)	0.43	0.37	0.49	0.05	-0.11	0.20
Vitamin E (mg)	1.27	1.09	1.45	0.11	-0.09	0.31
Vitamin B12 (mcg)	1.15	0.99	1.29	0.01	0.01	0.01
Potassium (mg)	2.90	1.99	3.80	0.24	-0.04	0.51
Sodium (mg)	4.06	3.44	4.67	-0.23	-0.42	-0.04
Phosphorus (mg)	3.07	2.93	3.22	0.01	0.00	0.01
Calcium (mg)	1.31	1.19	1.43	0.01	0.01	0.01
Iron (mg)	1.27	1.19	1.35	0.01	0.01	0.01
Magnesium (mg)	2.17	1.54	2.79	0.18	-0.10	0.45
Copper (mg)	0.38	0.32	0.45	0.01	-0.04	0.02
Zinc (mg)	1.07	0.84	1.30	0.01	-0.29	0.23
Selenium (mcg)	2.08	1.99	2.16	0.01	0.01	0.01
Energy (kcal)	3.45	2.48	4.42	0.01	-0.29	0.29

CI = confidence interval; RE = retinol equivalent.

α is the regression constant, λ is the slope of the regression line.

**Table 5:** Mean values of estimated, residual, constant, and adjusted macronutrients and micronutrients. Santo Antônio de Jesus, Bahia, Brazil, 2012

Nutrients	Estimated nutrient		Residual nutrient		Constant nutrient		Adjusted nutrient	
	FFQ	24hR	FFQ	24hR	FFQ	24hR	FFQ	24hR
Carbohydrate*	2.62	2.47	0.01	0.009	2.62	2.48	2.62	2.48
Protein*	1.95	1.89	0.03	-0.27	1.9	1.99	1.96	1.89
Fat*	1.74	1.95	0.02	0.03	1.75	1.95	1.75	1.87
Fibers*	1.48	1.15	-2.20	-0.01	1.48	1.15	1.48	1.15
Polyunsaturated fat*	1.11	0.70	0.01	-0.01	1.10	0.71	1.11	0.70
Monounsaturated fat	1.48	0.99	-0.54	0.84	1.48	1.89	1.48	1.89
Saturated fat	1.37	1.18	-0.01	0.01	1.37	1.19	1.37	1.19
Cholesterol	2.46	2.47	0.55	0.01	2.46	2.47	289.7	2.47
Vitamin A	3.46	5.55	0.01	1.09	3.46	1.36	3.45	4.91
Vitamin D*	3.58	1.10	0.03	-0.96	3.58	1.10	3.77	3.56
Vitamin C*	2.62	2.11	-0.62	0.91	2.62	2.11	1.1	2.11
Vitamin B3	1.45	1.33	-0.03	-0.62	1.46	1.33	1.46	1.32
Vitamin B1*	0.42	4.04	0.00	-0.97	0.43	4.04	0.43	3.06
Folate*	2.65	1.98	-0.67	-0.01	2.65	1.98	1.98	1.98
Vitamin B2*	0.45	3.36	0.001	0.95	0.45	3.36	0.46	4.31
Pantothenic acid*	0.88	5.77	-0.001	-0.99	0.88	5.77	0.88	4.78
Vitamin B6*	0.43	-0.05	0.03	-0.01	0.43	-0.04	0.43	-0.04
Vitamin E*	1.35	0.80	-0.001	0.00	1.36	0.80	1.35	0.80
Vitamin B12	1.19	1.17	0.00	-0.42	1.20	1.17	1.19	1.16
Potassium*	3.68	3.30	-0.0010	0.0012	3.67	3.30	3.67	3.30
Sodium*	3.33	3.30	0.00010	0.0012	3.32	3.30	3.30	3.30
Phosphorus	3.11	3.04	0.001	-0.007	3.11	3.04	3.12	3.04
Calcium	2.90	2.87	-1.62	1.06	2.91	-2.87	1.29	2.88
Iron*	1.29	1.20	-0.001	0.02	1.29	1.20	1.29	1.21
Magnesium*	2.56	2.24	0.00	-0.001	2.56	2.25	2.5	2.25
Copper*	0.40	2.43	-0.001	-0.47	0.40	2.43	0.40	1.95
Zinc*	1.05	0.83	-0.002	-0.002	1.05	0.83	1.04	0.84
Selenium*	2.09	1.77	0.001	-0.90	2.09	1.77	2.09	1.76

FFQ = food frequency questionnaire; 24hR = 24-hour recall.

* Values in logarithm.

## DISCUSSION

This study's results indicate that the validation and calibration of the FFQ increased the accuracy of the instrument when compared to the reference standard (24hR); that is, they better estimated the population values of energy availability, macronutrients, monounsaturated fat, cholesterol, vitamin B12, vitamin C, folate, sodium, iron, and calcium, allowing the estimates produced by the instruments to better reflect the actual consumption.^4^ Thus, this instrument can be used to associate feeding in the gestational period with maternal and fetal health.

Thus, the impact of applying statistical validation and calibration techniques adopted in this study was clearly observed by the change in the trend of agreement of the estimates of the values of crude, adjusted, and calibrated nutrients. Crude nutrients are values directly measured from the FFQ without any statistical treatment. Adjusted nutrients refer to those obtained after controlling for the effect of total energy available in the diet.^1^ Specifically, this adjustment minimizes the confounding effect of total energy.

This study's calibration brought the estimates obtained from the FFQ closer to those provided by the adopted reference method (24hR). Essentially, it minimized or eliminated biases, thereby more precisely measuring the dietary intake of the investigated pregnant women.

The crude food consumption estimates, calculated using the FFQ, overestimated the mean availability of energy and most nutrients, compared with the 24hR standard. After adjusting for energy, we observed that many values of the assessed nutrients decreased, possibly because the diet's total energy value artificially raised the estimated values from the 24hR. Thus, total energy was a confounding factor in the evaluated relationship. When total energy was controlled for in the equation, we noted that the other nutrients’ values decreased, possibly because the external variations affecting the increase in these values were removed. We observed a decrease in the values of the estimated correlations and statistical significance. A study conducted in Brazil on pregnant women reported similar results, characterized by declining values of nutrient correlation coefficients after adjusting for energy, indicating that energy can change the individual values of dietary nutrients.^8^

The results of this study confirm existing findings regarding the under- or over-estimation of consumption^1^ due to the assessment methods, indicating the need to calibrate these instruments to correct such errors.

Thus, calibration can shift estimates of dietary intake closer to the actuals, making the estimates more accurate and less biased.^22^ The adjustment or calibration factors (λ) of the nutrients found in this study ranged from −0.23 (sodium) to 0.24 (potassium), except for folate, whose correction factor was 1.00. In studies on dietary data calibration, the ideal is to have the estimated parameters for the intercept (α) close to zero and the estimated values for λ close to the unit.^23^

The calibration factors can be considered attenuated as they were smaller than the unit for most of the nutrients investigated. This result can be explained by the flattened slope effect, which implies an attenuated slope of the line (λ), generated by the control of several sources of bias (of information, in the reference instrument, in variations in the study period, and dietary calculations).^23,24^ Results available in the literature indicate a similar trend of attenuation in the values of the calibration factors, with different variations between 0.10 and 0.48;^10,25,26^ 0.50 and 0.70;^27,28^ and between −0.05 and 0.28,^23^ 0.4 and 0.9.^29^

The ideal method of diagnosing a population's food consumption should result in a nutrient distribution curve with zero mean and a standard deviation of 1. Obtaining these results would suggest that 95% of the assessed consumption would be similar to the population's consumption. This condition would indicate a lack of bias; that is, the mean intake captured by the instrument would be identical to the mean population's nutrient consumption as measured by the methodological instruments, if they were error-free. Reaching this level of perfection with a diagnostic instrument in any human health and nutritional situation could be unrealistic, considering the complex and multifaceted determination of people's living conditions.

However, no available method for evaluating food consumption meets these methodological conditions for qualifying it as the gold standard for evaluating food consumption; that is, all are subject to errors. These aspects also contribute to the low correlations found in this and other validation studies. This can be attributed to the limitations of the instruments (FFQ and 24hR) regarding intake estimates concerning the overestimated and underestimated portions consumed.^1,30,31^

Small correlations can also result from biased reporting, nutrient concentration differences among food lists and preparations in food composition tables used by food survey calculation software, and the use of the instruments’ differential measurement scale.^30,31^

Additional considerations regarding this study's adopted methodology should be highlighted. The absence of normal distribution for some variables indicated the need for logarithmic transformation of those with non-parametric distribution.^4^ Thus, some variables remained in their original form, while others were converted into logarithms, possibly limiting the reader's understanding.

Reapplying Pearson's correlation test, after the calibration phase, between the calibrated nutrients from the FFQ and the adjusted nutrients from the 24hR made the method more accurate in estimating the availability of energy and other nutrients from population values. Thus, the statistical analyses were consistent with the validation studies of FFQ in pregnant women, with *Pearson* or *Spearman's* correlation coefficient for validation purposes.^32^

Some food consumption studies finalized the validation of the questionnaire at this methodological stage,^28,33,34^ while others did so after the calibration of nutrients was adjusted for energy.^35–37^ However, in this study, besides validation and calibration, a new correlation test was performed between the calibrated nutrient values and those obtained by the reference method. This stage of verifying the final correlations, theoretically supported by the statistical assumptions of the test application, aimed to verify the agreement between the calibrated estimates obtained by the method under test versus the reference method and is a differentiated step for consistency.

This step reinforces that this study's results did not occur by chance, given the methodological rigor adopted throughout, to allow for the attenuation of the limits inherent in the elaboration and application of the instruments, and the adoption of the analysis of food consumption information. Even so, the lack of standardization in the collection and analysis of food consumption data limits the impact and expectation of robust results that could be produced using validation analysis and instrument calibration.^1,23^

In assessing food consumption, the appropriateness of applying more than one instrument to record consumption or more than one recall compared with the FFQ should also be considered. This recommendation is based on the observation that studies that adopted four recalls registered low correlation coefficients for some nutrients.^14^

However, the calibration technique, as a statistical instrument, and the validation analysis this study used can minimize the adoption of only one 24hR compared with the instrument under test^14^ and make the methodological model statistically more robust. Using only one 24hR instrument may not necessarily result in a significant difference in the variation of consumption reports, and this study's results were close to the population's consumption.

This study has several limitations. The assessment of food consumption depended on the participants’ memory and ability to report the measurements and portions consumed. Food records, especially those using direct weighing, are better instruments for correctly estimating food consumption. Nevertheless, there could be better instruments for the sample, as study's participant have a low level of education.^38^ In future studies, food intake for fewer days should be evaluated. In this study, the greater the number of days assessed by the standard diet instrument, the smaller the error inherent in the variability of interindividual consumption.^38^ Generally, despite overestimating food intake, the FFQ showed good calibration and agreement with the 24hR and may be used in clinical practice to assess the food intake of pregnant women.

## CONCLUSION

This study contributes to nutritional epidemiology by expanding and improving knowledge regarding research techniques and instruments that minimize possible errors in measuring food consumption, a variable that is strongly influenced by numerous biological, social, cultural, and economic factors. The validated and calibrated FFQ can globally evaluate a pregnant woman's diet and can be used in a complementary way to specific nutrient instruments for this group.^38^ We can conclude that the FFQ used in this study can be employed in other epidemiological investigations to assess the food consumption of pregnant women with similar socioeconomic and demographic characteristics.
